# Prospective association between depressive symptoms and hip fracture and fall among middle-aged and older Chinese individuals

**DOI:** 10.1186/s12888-022-03906-2

**Published:** 2022-04-12

**Authors:** Chunsu Zhu, Hongyu Yu, Zhiwei Lian, Jianmin Wang

**Affiliations:** Fujian Medical University Cancer Hospital, Fujian Cancer Hospital, No. 420, Fuma Road, Jinan District, Fuzhou, 350014 China

**Keywords:** Depressive symptoms, Hip fracture, Falls, Cohort study, Middle-aged, Older adults

## Abstract

**Background:**

The effect of depressive symptoms on hip fracture (HF) and falls among the Chinese population is unclear. This study aims to examine the prospective association between depressive symptoms and HF as well as fall accidents in a nationally representative Chinese population.

**Methods:**

We used data from 10,596 participants in the Chinese Health and Retirement Longitudinal Study (from 2011 to 2018) who were aged ≥45 years and had no HFs at baseline. Depressive symptoms were assessed using the 10-item version of the Center for Epidemiological Studied Depression scale (cutoff for distinguishing high versus low at ≥12). Logistic regression analyses adjusted for demographic characteristics, lifestyle factors and physical comorbidities were performed.

**Results:**

For the analysis of baseline depressive symptoms and HF, 399 (3.8%) participants reported HF accidents in the following 7-year period. Individuals with elevated depressive symptoms at baseline experienced a markedly higher HF risk (odds ratio [OR] = 1.33, 95% confidence interval [CI] = 1.06–1.67) than those without elevated depressive symptoms, after adjusting for a wide range of potential confounders. For the analysis of baseline depressive symptoms and falls, 3974 (37.5%) experienced fall accidents during the follow-up. The presence of elevated depressive symptoms was independently associated with an increased risk of fall events (OR = 1.21, 95% CI = 1.10–1.33). These associations were consistent across multiple characteristics.

**Conclusions:**

In conclusion, elevated depressive symptoms were associated with an increased risk of HF and falls, which may have considerable clinical and preventive implications.

**Supplementary Information:**

The online version contains supplementary material available at 10.1186/s12888-022-03906-2.

## Introduction

Hip fracture (HF) is currently a major public health issue both worldwide and in China because it has a high prevalence and incidence [1, 2], and it causes increased mortality rate [3], disability rates and medical costs [4–6]. Studies have shown that nearly 20–30% of patients die in the year following HF [1, 7]; half of those who fortunately survive lose functional independence, and approximately 30% eventually need long-term care [8]. It was predicted that the number of HFs worldwide will increase by 3.6-fold by 2050, and approximately 50% of these HFs will occur in Asia, especially in China [9]. The incidence of HF increases due to population aging, since the incidence of HF is closely associated with increasing age, particularly after the age of 60 years [10, 11]. Eighteen percent of the global population lives in mainland China, and this population is rapidly aging. In 2019, the number of individuals aged 65 years and above reached 164.5 million, and the number is predicted to reach nearly 365 million by 2050 [12]. Although a survey showed that the incidence of HFs among individuals aged 55 years and over in China remained stable from 2012 to 2016, the absolute number of HFs and related hospitalization costs have increased rapidly [2]. Falls are the leading cause of HF [13]; they are also common in older adults [14], and are related to considerable morbidity, reduced quality of life and increased healthcare expenses [15]. Given the significant morbidity, mortality, and healthcare burdens associated with HF and falls, understanding and preventing hip fracture and falls is an important priority.

Depression is one of the most common psychiatric disorders, affecting almost 3.8% of the population worldwide [16]. In China, a national survey reported that approximately 30% of men and 43% of women aged 45 years and above experienced depressive symptoms [17]. Depression may increase HF risk through several potential pathways, including lowered bone mineral density (BMD), unhealthy behaviors (e.g., poor diet, smoking, inactivity, poor adherence to medical advice), decreased cognitive function and psychomotor retardation [18, 19]. At present, emerging cross-sectional and prospective studies have assessed the relationship between depression and the risk of HF or falls [20–25], but these were mainly conducted in developed regions (e.g., the US) with inconsistent results [20]. More investigations conducted at various national income levels, socioeconomic statuses and demographic features with standardized study methods and well-designed confounding adjustments are warranted to confirm the association. A meta-analysis of 16 cohort studies showed that the association between depression and the risk for total fracture was different among diverse countries, and the pooled hazard ratio for total fracture was higher in Europe than in America [26]. Therefore, the findings from other countries may not be generalizable to China. However, to the best of our knowledge, no study has yet investigated the associations between depressive symptoms and HF in mainland China. Considering the high disease burden of HF, if confirmed, the association between depressive symptoms and HF may open new directions for HF prevention. Consequently, the purpose of this study is twofold: First, we assessed the effect of baseline depressive symptoms on the risk of HF and falls among middle-aged and older Chinese individuals. Second, we compared the risk of HF and falls in different subgroups to identify vulnerable populations for prevention.

## Methods

This study followed the Strengthening the Reporting of Observational Studies in Epidemiology (STROBE) guidelines.

### Study population

The data were derived from the China Health and Retirement Longitudinal Study (CHARLS), a nationally representative cohort study of residents aged 45 and older. A detailed description of the project has been provided elsewhere [27]. In brief, to ensure sample representativeness, a multistage stratified probability-proportional-to-size sample technique was used. At baseline, a total of 17,708 individuals in 10,257 households were recruited from June 2011 to March 2012, covering 28 provinces, 150 countries/districts, and 450 villages/urban communities in mainland China. Demographic and lifestyle factors and health-related information were collected by well-trained interviewers, using a standardized questionnaire. All participants were reinterviewed every 2 years after the baseline survey.

**Fig. 1:**
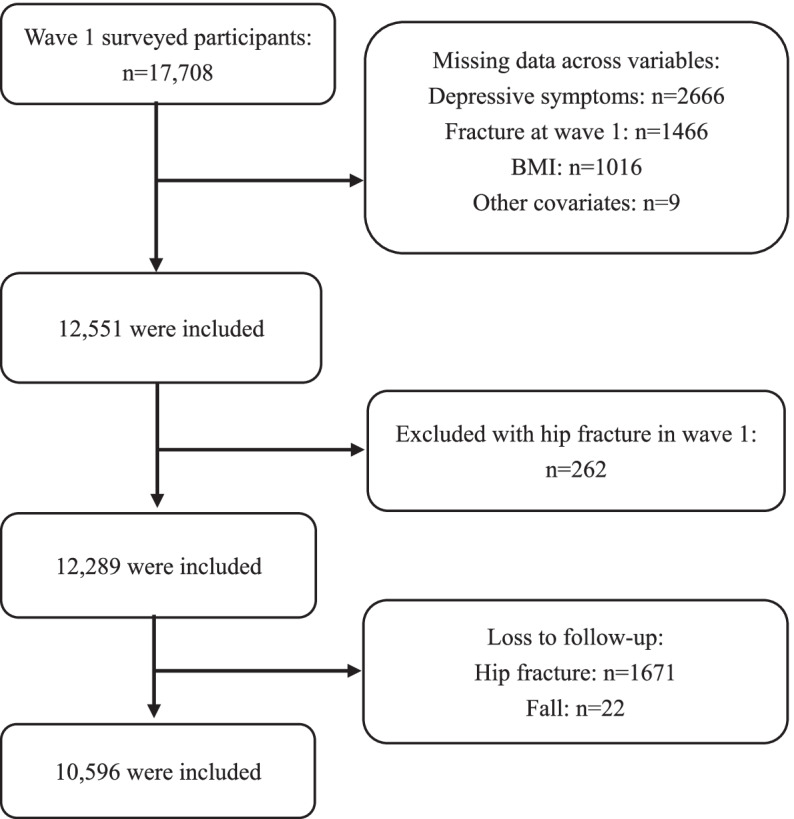
The selection process of study participants. BMI: body mass index; wave 1: the baseline survey of the China Health and Retirement Longitudinal Study (CHARLS) conducted in 2011

This current study reports data spanning 8 years, from wave 1 in 2011 to wave 4 in 2018. A flow diagram of how the sample was derived is provided in Fig. 1. We excluded those with a history of hip fracture at baseline, with missing values on depressive symptoms (the missingness frequency of each depressive symptom was provided in Supplementary Table S1), HF, falls, and other covariates. Finally, 10,596 individuals were included for further analysis. We compared the baseline characteristics for all participants, those included and those excluded from the current analyses (Supplementary Table S2). Due to the large sample size, the tests for several characteristics between those included and excluded participants were statistically significant. Except for age and body mass index (BMI), other characteristics were largely comparable between those included and excluded participants. The participants included in the current analysis were somewhat younger and less likely to be underweight than those excluded. Particular reasons for those loss to- follow-up may include death, population migration or refusal to participate in the study.

### Depressive symptoms

Depressive symptoms were measured using the 10-item version of the Center for Epidemiological Studied Depression scale (CESD-10), which is widely used to measure depressive symptoms in population-based studies. The CESD-10 consists of ten items: (1) bothered by things, (2) had trouble concentrating, (3) feel depressed, (4) everything was an effort, (5) felt hopeful, (6) felt fearful, (7) sleep was restless, (8) felt happy, (9) felt lonely, and (10) could not get going. Each item was scored as 0, 1, 2, or 3 scores, and positive items were reverse scored. The total depressive symptoms score was calculated by summing scores across all items, ranging from 0 to 30. A total score of at least 12 was used as the cutoff for elevated depressive symptoms [28]. Previous studies have shown that the CESD-10 has high reliability and validity for older Chinese individuals [29].

### Hip fracture and fall

The primary outcome of interest in this study is the first HF. Information on HF accidents was based on self-reports according to the following two questions: “Have you ever fractured your hip?” and “Have you fractured your hip since the last interview?”. The answer was categorized as “yes” or “no”. The secondary outcome of interest is fall. Participants were asked “Have you fallen down in the last two years?”. The response was classified as “yes” or “no”.

### Covariates

At baseline, information on demographic characteristics, lifestyles, and health-related factors was collected by qualified interviewers using a structured questionnaire, including age, sex, place of residence, marital status, and education (below high school, high school and above). Marital status was classified as married and others (never married, separated, divorced, and widowed). Lifestyle factors included self-reported smoking status (never, current, quit), drinking frequency (never, rarely, often), and sleep duration (< 7 h, ≥7 h). BMI was calculated as the weight in kilograms divided by the square of height in meters, and was classified into four categories according to the guidelines for Chinese people [30, 31]: underweight (< 18.5 kg/m^2^), normal weight (≥ 18.5 kg/m^2^ and < 24.0 kg/m^2^), overweight (≥ 24.0 kg/m2 and < 28.0 kg/m^2^) and obese (≥ 28.0 kg/m^2^). Chronic physical diseases included hypertension, dyslipidemia, diabetes, heart problems (including heart attack, coronary heart disease, angina, congestive heart failure, or other heart problems), chronic lung diseases (including chronic bronchitis, emphysema, excluding cancers or tumors), liver diseases (except for tumors or cancers), cancers, stroke, kidney disease (except for cancers or tumors), stomach or other digestive disease (except for cancers or tumors), and arthritis or rheumatism. Physical comorbidities were classified as 0, 1, 2, or ≥ 3 based on the number of chronic diseases.

### Statistical analysis

All analyses were carried out using Statistical Product and Service Solution (SPSS) 26.0, and forest plots were drawn with R software (package forestplot). Baseline characteristics are shown as numbers and proportions for categorical data, and means and standard deviations (SD) for continuous data, according to depressive symptoms. Comparison analyses between groups were conducted using independent t tests for continuous variables and chi-square tests for categorical variables. A two-sided *p* value < 0.05 was considered statistically significant. Logistic regression was performed to assess the relationship between baseline depressive symptoms (modeled continuously, dichotomously and quintiles) and incident HF reported between wave 1 and wave 4, adjusting for covariates. We first controlled for age and sex in model 1. Then place of residence, marital status, education, smoking, drinking, sleep duration, and history of fall accidents were added. In the fully adjusted model, BMI and physical comorbidities were also adjusted. To further evaluate the association between the severity of depressive symptoms and incident HF events, depressive symptom scores were split into quintiles and then included in the logistic regression models with quintile 1 as the referent. To examine the longitudinal relationship between baseline depressive symptoms and the risk of falling, similar series of logistic models were fitted. Logistic regression was selected for two important reasons. First, creating a time variable on the basis of the year of first reporting is arbitrary and is of little clinical value because participants were assessed biennially. Second, we were unable to create a time variable using month and year of accident onset because these data were unavailable.

*P* values for interaction were calculated for all aforementioned covariates, using interaction terms. Then subgroup analyses were conducted to examine whether the associations between depressive symptoms and HF events were moderated by these factors. To reduce the false discovery rate, a more stringent *p* value was adopted for subgroup and interaction analyses (two-sided *p* value < 0.01). Three sensitivity analyses were performed as follows: (1) excluding participants currently using antidepressants since previous studies have suggested that the intake of antidepressants may increase the risk of HF [32]; (2) excluding those aged 80 and older at baseline (more likely to fall down and experience fractures); and (3) excluding those who reported HF in the first follow-up wave (wave 2); and repeated the analyses.

## Results

A total of 10,596 adults were included in the analyses. The mean (SD) age at baseline was 58.3 (9.2) years; 5043(47.6%) were men. Table 1 presents the characteristics of the participants. A total of 2865 (27.0%) participants had elevated depressive symptoms (CESD-10 ≥ 12). Participants with depressive symptoms were more likely to be older (59.7 vs. 57.9, *p* < 0.001), be female (63.3% vs. 48.4%, *p* < 0.001), living in rural areas (rural residence, 73.1% vs. 60.0%, *p* < 0.001), have lower education (below high school, 94.2% vs. 84.3%, *p* < 0.001), be unmarried (17.8% vs. 8.8%, *p* < 0.001), be never smokers (65.5% vs. 58.9%, *p* < 0.001), be never drinkers (66.6% vs. 59.5%, *p* < 0.001), be with comorbidities (have ≥3 comorbidities, 36.2% vs. 20.9%, *p* < 0.001) and have a history of falls (23.8% vs. 12.4%, *p* < 0.001).

**Table 1:** Baseline characteristics of 10,596 participants aged ≥45 years according to depressive symptoms

Characteristics	Total sample (*N* = 10,596)	Depressive symptoms		*P* Value
		No (*n* = 7731)	Yes (*n* = 2865)	
Age, mean(SD), y	58.3(9.2)	57.9(9.2)	59.4(9.3)	< 0.001
Age categories, y, n(%)				< 0.001
45–59	6255(59.0)	4733(61.2)	1522(53.1)	
60–69	2966(28.0)	2050(26.5)	916(32.0)	
≥ 70	1375(13.0)	948(12.3)	427(14.9)	
Sex, n(%)				< 0.001
Men	5043(47.6)	3992(51.6)	1051(36.7)	
Women	5553(52.4)	3739(48.4)	1814(63.3)	
Residency, n(%)				< 0.001
Rural	6733(63.5)	4638(60.0)	2095(73.1)	
Urban	3863(36.5)	3093(40.0)	770(26.9)	
Education, n(%)				< 0.001
Below high school	9220(87.0)	6521(84.3)	2699(94.2)	
High school and above	1376(13.0)	1210(15.7)	166(5.8)	
Marital status, n(%)				< 0.001
Married	9404(88.8)	7050(91.2)	2354(82.2)	
Others	1192(11.2)	681(8.8)	511(17.8)	
Smoking status, n(%)				< 0.001
Never	6429(60.7)	4553(58.9)	1876(65.5)	
Current	3255(30.7)	2484(32.1)	771(26.9)	
Quit	912(8.6)	694(9.0)	218(7.6)	
Drinking frequency, n(%)				< 0.001
Never	6506(61.4)	4599(59.5)	1907(66.6)	
Rarely	849(8.0)	635(8.2)	214(7.5)	
Often	3241(30.6)	2497(32.3)	744(26.0)	
BMI, kg/m^2^, n(%)				< 0.001
Normal weight	5646(53.3)	4058(52.5)	1588(55.4)	
Underweight	931(8.8)	649(8.4)	282(9.8)	
Overweight	2872(27.1)	2155(27.9)	717(25.0)	
Obese	1147(10.8)	869(11.2)	278(9.7)	
Comorbidity, n(%)				< 0.001
0	2098(19.8)	1731(22.4)	367(12.8)	
1	3168(29.9)	2440(31.6)	728(25.4)	
2	2678(25.3)	1946(25.2)	732(25.5)	
≥ 3	2652(25.0)	1614(20.9)	1038(36.2)	
Sleep duration, hours				< 0.001
< 7	5260(49.6)	3417(44.2)	1843(64.3)	
≥ 7	5336(50.4)	4314(55.8)	1022(35.7)	
History of falls, n(%)	1640(15.5)	958(12.4)	682(23.8)	< 0.001

T tests were used for continuous variables, and chi-square tests were used for categorical variables

*SD* Standard deviation, *BMI* Body mass index

Of all participants who were free of HF at baseline, 399 (3.8%) experienced HF incidents during the follow-up period from 2011 to 2018. Table 2 shows the binary logistic regression analyses associations between depressive symptoms and HF. In model 3, after adjusting for potential cofounders including age, sex, residency, education, marital status, smoking status, drinking status, sleep duration, history of falls, BMI and physical comorbidities, the risk of HF for a 1-unit increase in the CESD-10 score (modeled as a continuous variable) was 1.03 (95% confidence interval [CI] = 1.01–1.04), and baseline elevated depressive symptoms were independently associated with a 33% increased risk of HF (odds ratio [OR] = 1.33, 95% CI = 1.06–1.67). When the total CESD-10 score was modeled as quintiles (Table 2), similar results were found. By comparing quintile 5 with quintile 1, the adjusted OR was 1.67 (95% CI =1.19–2.35) in the fully adjusted model. In total, 3974 (37.5%) experienced fall incidents during the follow-up period. Elevated depressive symptoms were associated with a 21% increased risk of fall events (OR = 1.21, 95% CI = 1.10–1.33) after full adjustment in model 3 (Table 2). After controlling for potential confounders (in model 3), the OR of fall incidence for one-unit increase in the CESD-10 score was 1.03 (95% CI = 1.02–1.03). Comparing quintile 5 with quintile 1, the adjusted risk of fall incidents was 1.48 (95% CI = 1.29–1.69) (Table 2).

**Table 2:** Association between baseline depressive symptoms and hip fracture and fall risk

Outcome	OR(95% CI)		
	Model 1	Model 2	Model 3
**Hip fracture**			
Depressive symptom scores, continuous	1.03(1.01,1.05)	1.03(1.01,1.04)	1.03(1.01,1.04)
Depressive symptoms status			
No	Reference	Reference	Reference
Yes	1.40(1.13,1.74)	1.35(1.08,1.68)	1.33(1.06,1.67)
Depressive symptom scores, quintile			
1(0–3)	Reference	Reference	Reference
2(4–5)	1.35(0.94,1.94)	1.33(0.92,1.92)	1.33(0.92,1.91)
3(6–9)	1.53(1.13,2.07)	1.49(1.10,2.03)	1.48(1.09,2.02)
4(10–14)	1.57(1.14,2.16)	1.52(1.10,2.10)	1.51(1.09,2.09)
5(15–30)	1.78(1.29,2.45)	1.69(1.21,2.36)	1.67(1.19,2.35)
**Fall**			
Depressive symptom scores, continuous	1.03(1.03,1.04)	1.03(1.02,1.04)	1.03(1.02,1.03)
Depressive symptoms status			
No	Reference	Reference	Reference
Yes	1.38(1.26,1.50)	1.26(1.15,1.38)	1.21(1.10,1.33)
Depressive symptom scores, quintile			
1(0–3)	Reference	Reference	Reference
2(4–5)	1.15(1.00,1.32)	1.11(0.97,1.28)	1.10(0.96,1.27)
3(6–9)	1.50(1.34,1.68)	1.44(1.28,1.61)	1.41(1.26,1.58)
4(10–14)	1.73(1.53,1.95)	1.61(1.42,1.82)	1.56(1.37,1.76)
5(15–30)	1.75(1.55,2.00)	1.56(1.37,1.77)	1.48(1.29,1.69)

Model 1 was adjusted for age and sex; Model 2 was adjusted for age, sex, residency, education, marital status, smoking status, drinking status, sleep duration and history of falls; Model 3 was further adjusted for BMI and physical comorbidities

Stratification analyses and interactions for the association between depressive symptoms and HF and falls were then conducted for all aforementioned variables (Supplementary Figs. S1 and S2). No significant interactions were detected for all variables (all p-interaction > 0.01). In the sensitivity analyses (Table S2), the results did not significantly change after excluding participants currently taking antidepressants (HF, OR = 1.34, 95% CI = 1.07–1.68; fall, OR = 1.22, 95% CI = 1.11–1.34). Similar results were found when excluding those who were aged ≥80 years or those who reported HF accidents in the first follow-up wave.

## Discussion

In this nationally representative cohort study of 10,596 adults aged 45 years and older with 7 years of follow-up, we found that depressive symptoms were associated with a 33% increased risk for HF and a 21% increased risk for falls after full adjustment, including a history of prior falls. The risk of HF increased in parallel with the severity of depressive symptoms. In addition, these associations remained consistent across different characteristics.

Our results showed that there is a significant association between depressive symptoms and HF and falls among middle-aged and older individuals, which is in line with previous studies [33–35]. Consistent with previous studies, our findings further confirmed the positive relationship between depressive symptoms and HF risk in the Chinese population. However, we found that participants with elevated depressive symptoms had a 1.33 higher risk of HF than those without, which was slightly different from the results of previous studies. A recent meta-analysis of 10 cohort studies reported that depressive symptoms were associated with a 21% increased risk of HF (pooled hazards ratio (HR) =1.21, 95% CI = 1.11–1.31) [20]. The National Health and Nutrition Examination Survey of individuals aged 25 through 74 showed that elevated depressive symptoms were marginally associated with an increased risk of HF (adjusted HR = 1.70, 95% CI = 0.99–2.91) [23]. These differences might be attributed to the widely varied characteristics of the study populations (e.g., age, sex composition, ethnicity or race), different severities of depressive symptoms, different study designs (e.g., follow-up duration, sample size), and different statistical analysis methods (e.g., adjusted confounds). For instance, the studies included in the abovementioned meta-analysis abovementioned were mainly conducted in high-income areas, such as those in Europe and North America [20], while our findings were derived from a Chinese population. A meta-analysis suggested that the relationship between depressive symptoms and fractures is inconsistent among continents with different income levels, which might support our speculations [26]. In addition, an investigation conducted in Taiwan reported that patients with major depressive disorder had a higher incidence of HF than those without, but those with less severe depressive disorder did not have a significantly higher risk than those with no depressive symptoms [21]. Our results found a stronger association in those with higher depressive symptom scores might also help explain the variability to some extent.

The underlying mechanism between depression and the increased risk of HF has not been fully elucidated, but may be due to the following: First, depression is related to some health-related behaviors, such as unhealthy diet [36], smoking [37], alcohol consumption, sedentary lifestyles and poor adherence to medical advice [38, 39], which may affect bone metabolism [40]. Second, depression may impact bone formation and bone resorption by several biological pathways. On the one hand, depression is associated with increased levels of inflammatory cytokines (e.g., interleukin-2 and interleukin-6), and studies have suggested that these inflammatory markers are associated with decreased BMD [41]. On the other hand, depression is linked to low vitamin D levels, hypercortisolemia and alteration of other hormone concentrations (e.g., estrogen, testosterone, insulin growth factors), which are key regulators in bone formation and bone resorption [42, 43]. Third, depression is associated with a variety of physical comorbidities (e.g., stroke) that can increase the risk of falls and HF [44]. In addition, depression is associated with cognitive function decline, psychomotor retardation and judgment impairment, which may affect a patient’s gait, balance and safety precautions against falls [45, 46]. Both decreased BMD and increased possibility of falling are likely to increase the risk of HF. Finally, previous studies have suggested that antidepressants may increase the risk of HF [47]. However, the antidepressant prescription or use might be merely a symbol of the severity of depression, and in our sensitivity analysis, we excluded individuals who reported the use of antidepressants. The results showed robustness.

To the best of our knowledge, this study is the first to examine the association between depressive symptoms and the risk of HF in a mainland Chinese population. The main strengths included the large sample size, prospective design and adjustments for a wide range of cofounders. However, some limitations should be noted. First, HF and falls were self-reported. Although this is common in most large epidemiological studies, misclassification bias is inevitable. Nevertheless, a previous investigation tested the difference in falls for people with different levels of balance, and the results showed that falls were significantly associated with balance assessment; this suggests that self-reported fall results have enough validity [33]. In fact, it is difficult to collect information on falls through objective measures (e.g., fall calendar) in such a large epidemiological study, and many other similar large investigations rely on self-reported questionnaires (e.g., the Health and Retirement Study in the USA and the Survey of Health and Retirement in Europe). Despite of this, we had to admit that the fall incidence in our study was still relatively underestimated due to recall bias, which should be explained cautiously. In addition, HF accidents are serious conditions, and patients generally seek medical examinations as well as treatments from doctors, which should not have impacted our conclusions. Second, depressive symptoms were measured using the CESD-10 rather than clinical diagnosis, which may underestimate the effect of depressive symptoms on HF. However, our findings do suggest that adults with elevated depressive symptoms suffer from a higher risk of HF and falls, highlighting the potential public health implications of screening people with depressive symptoms to prevent HF and fall accidents among middle-aged and older populations. Third, as mentioned before, some psychiatric medications, such as selective serotonin reuptake inhibitors (SSRIs) and tricyclic antidepressants (TCAs), are associated with the risk of falls or fractures [32, 48]; however, information on specific antidepressant medications was unavailable in CHARLS, and only a small number of participants (only 49 individuals) reported the use of medications to treat depressive symptoms, which is a major limitation of this study. Fourth, compared with those excluded from our analysis, those included represented a younger subset of individuals and were less likely to be underweight, which may have led us to underestimate some of the effect. In addition, due to the presence of subclinical or clinical diseases (e.g., bone loss, structural damage, calcium or vitamin D deficiency), there might be some potential reverse causation bias. To address this issue, we excluded those aged 80 and older, and those who experienced fractures between baseline and the first follow-up wave, and repeated analyses remained robust. Finally, the CHARLS included extensive data on demographic characteristics, lifestyle factors and chronic physical diseases, enabling us to adjust a series of confounding factors, yet it was not possible to collect information on all potential confounders. For instance, physical activity and physical capacity (e.g., muscle strength, gait, speed) were not controlled, and we were unable to conduct a survival analysis; thus residual confounding may still be present.

In conclusion, the findings obtained from this large-scale cohort study further confirmed that depressive symptoms are significantly associated with an increased risk of HF and falls among middle-aged and older Chinese individuals. Given the high prevalence of depressive symptoms and increasing burden of HF in China, these findings have important public health implications, especially with the increasing aging of the population. Effective management of depressive symptoms may be applied in the prevention of HF and falls among middle-aged and older people. Future studies are needed to investigate the potential mechanisms underlying the association between depressive symptoms and HF and falls.

## Supplementary Information

**Additional file 1: Table S1.** The missingness frequency of each CESD-10 item. **Table S2.** Baseline characteristics of all participants, those included and excluded in our analyses. **Figure S1.** Subgroup analysis of the association between depressive symptoms and hip fracture. Depressive symptom scores were added as a continuous variable. Models were adjusted for age, sex, residence, education, marital status, smoking status, drinking status, sleep duration, history of falls, BMI and physical comorbidities as appropriate. **Figure S2.** Subgroup analysis of the association between depressive symptoms and fall. Depressive symptom scores were added as a continuous variable. Models were adjusted for age, sex, residence, education, marital status, smoking status, drinking status, sleep duration, history of falls, BMI and physical comorbidities as appropriate. **Table S3.** Sensitivity analyses for the association between depressive symptoms and hip fracture and fall.

## Data Availability

The data that support the findings of this study are available from the China Health and Retirement Longitudinal Study (CHARLS) group, but restrictions apply to the availability of these data, which were used under license for the current study, and so are not publicly available. Data are however available from the authors upon reasonable request and with permission of CHARLS.

## Abbreviations

HF: Hip fracture
CESD-10: 10-item version of the Center for Epidemiological Studied Depression scale
OR: Odds ratio
CI: Confidence interval
BMD: Bone mineral density
CHARLS: China Health and Retirement Longitudinal Study
BMI: Body mass index
SPSS: Statistical Product and Service Solution
SD: Standard deviations
HR: Hazard ratio
SSRIs: Selective serotonin reuptake inhibitors
TCAs: Tricyclic antidepressants
