# Supplementary material for: Prospective association between depressive symptoms and hip fracture and fall among middle-aged and older Chinese individuals
Source: BMC Psychiatry. 2022 Apr 12;22:258. doi: 10.1186/s12888-022-03906-2 (PMC9004028; doi:10.1186/s12888-022-03906-2)
Supplement: Supplementary file 1 — Additional file 1: Table S1. The missingness frequency of each CESD-10 item. Table S2. Baseline characteristics of all participants, those included and excluded in our analyses. Figure S1. Subgroup analysis of the association between depressive symptoms and hip fracture. Depressive symptom scores were added as a continuous variable. Models were adjusted for age, sex, residence, education, marital status, smoking status, drinking status, sleep duration, history of falls, BMI and physical comorbidities as appropriate. Figure S2. Subgroup analysis of the association between depressive symptoms and fall. Depressive symptom scores were added as a continuous variable. Models were adjusted for age, sex, residence, education, marital status, smoking status, drinking status, sleep duration, history of falls, BMI and physical comorbidities as appropriate. Table S3. Sensitivity analyses for the association between depressive symptoms and hip fracture and fall. [file 12888_2022_3906_MOESM1_ESM.docx]

Table S1. The missingness frequency of each CESD-10 item

| **Item** | **Content** | **Missingness, n (%)** |
| --- | --- | --- |
| 1 | Bothered by things | 1787(10.1) |
| 2 | Had trouble concentrating | 1883(10.6) |
| 3 | Felt depressed | 1848(10.4) |
| 4 | Everything was an effort | 1818(10.3) |
| 5 | Felt hopeful | 2077(11.7) |
| 6 | Felt fearful | 1736(9.8) |
| 7 | Restless sleep | 1717(9.7) |
| 8 | Felt happy | 1760(9.9) |
| 9 | Felt lonely | 1784(10.1) |
| 10 | Could not get going | 1822(10.3) |

Table S2. Baseline characteristics of all participants, those included and excluded in our analyses

| Characteristics | All participants (n=17,708) | Included  participants (n=10,596) | Excluded  participants  (n=7112) | P value* |
| --- | --- | --- | --- | --- |
| Age, mean | 59.2 | 58.3 | 60.6 | <0.001 |
| Age categories (%) |  |  |  | <0.001 |
| 45-59 | 56.2 | 59.0 | 52.1 |  |
| 60-69 | 27.1 | 28.0 | 25.7 |  |
| ≥70 | 16.7 | 13.0 | 22.2 |  |
| Sex (%) |  |  |  | 0.294 |
| Male | 48.0 | 47.6 | 48.4 |  |
| Female | 52.0 | 52.4 | 51.6 |  |
| Place of residence (%) |  |  |  | <0.001 |
| Rural | 59.5 | 63.5 | 53.5 |  |
| Urban | 40.5 | 36.5 | 46.5 |  |
| Marital status (%) |  |  |  | <0.001 |
| Married | 87.3 | 87.0 | 84.9 |  |
| Others | 12.7 | 13.0 | 15.1 |  |
| Education (%) |  |  |  | 0.197 |
| Below high school | 87.2 | 88.8 | 87.7 |  |
| High school and above | 12.8 | 11.2 | 12.3 |  |
| Smoking status, (%) |  |  |  | 0.008 |
| Never | 60.4 | 60.7 | 60.0 |  |
| Current | 31.4 | 30.7 | 32.4 |  |
| Quit | 8.2 | 8.6 | 7.6 |  |
| Drinking frequency, (%) |  |  |  | 0.606 |
| Never | 61.1 | 61.4 | 60.7 |  |
| Rarely | 8.1 | 8.0 | 8.3 |  |
| Often | 30.8 | 30.6 | 31.0 |  |
| BMI, kg/m^2^, (%) |  |  |  | <0.001 |
| Normal weight | 52.8 | 53.3 | 51.8 |  |
| Underweight | 11.4 | 8.8 | 17.3 |  |
| Overweight | 25.7 | 27.1 | 22.6 |  |
| Obese | 10.1 | 10.8 | 8.3 |  |
| Comorbidity, (%) |  |  |  | <0.001 |
| 0 | 22.5 | 19.8 | 26.4 |  |
| 1 | 29.3 | 29.9 | 28.3 |  |
| 2 | 23.9 | 25.3 | 22.0 |  |
| ≥3 | 24.3 | 25.0 | 23.3 |  |
| Sleep duration, hours |  |  |  | 0.010 |
| <7 | 50.4 | 49.6 | 51.8 |  |
| ≥7 | 49.6 | 50.4 | 48.2 |  |
| History of fall, (%) | 15.7 | 15.5 | 16.1 | 0.295 |
| Depressive symptom scores, continuous(mean) | 8.4 | 8.7 | 8.3 | 0.001 |
| Depressive symptoms status (%) |  |  |  | <0.001 |
| No | 71.9 | 73.0 | 69.6 |  |
| Yes | 28.1 | 27.0 | 30.4 |  |
| Depressive symptom scores, quintile (%) |  |  |  | 0.004 |
| 1(0-3） | 26.4 | 26.9 | 25.2 |  |
| 2(4-5) | 13.0 | 13.4 | 12.2 |  |
| 3(6-9) | 23.5 | 23.5 | 23.7 |  |
| 4(10-14) | 19.5 | 19.2 | 20.0 |  |
| 5(15-30) | 17.6 | 17.0 | 18.9 |  |

*Comparison between those included and excluded in our study.


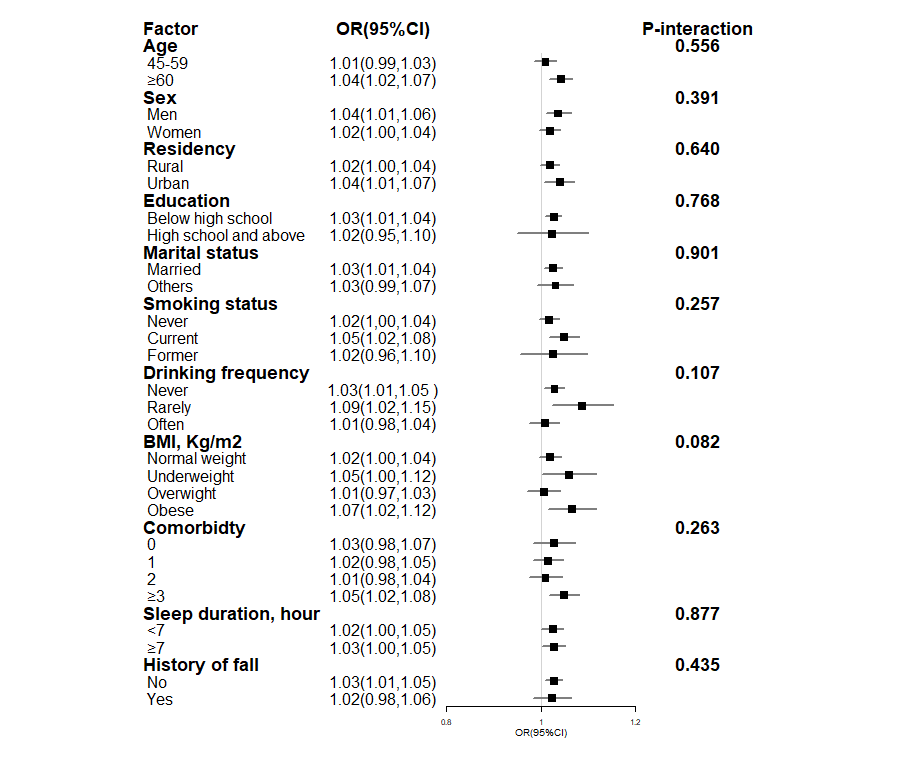


Figure S1. Subgroup analysis of the association between depressive symptoms and hip fracture. Depressive symptom scores were added as a continuous variable. Models were adjusted for age, sex, residence, education, marital status, smoking status, drinking status, sleep duration, history of falls, BMI and physical comorbidities as appropriate.


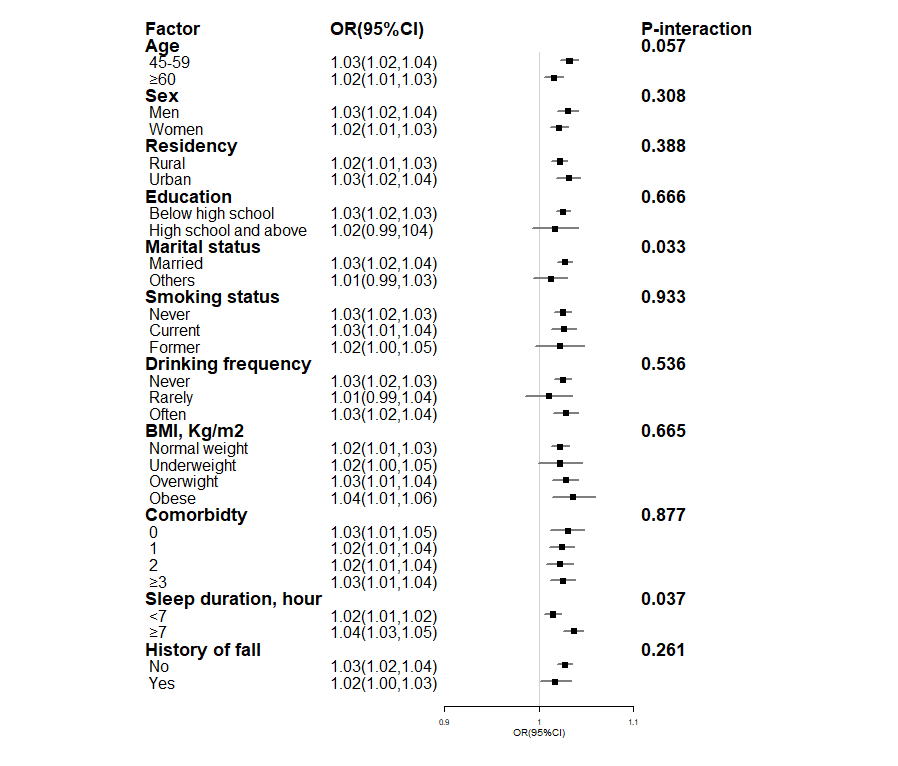


Figure S2. Subgroup analysis of the association between depressive symptoms and fall. Depressive symptom scores were added as a continuous variable. Models were adjusted for age, sex, residence, education, marital status, smoking status, drinking status, sleep duration, history of falls, BMI and physical comorbidities as appropriate.

Table S3. Sensitivity analyses for the association between depressive symptoms and hip fracture and fall

| **Outcome** | **OR(95%CI)** | | |
| --- | --- | --- | --- |
|  | Sensitivity analysis 1 | Sensitivity analysis 2 | Sensitivity analysis 3 |
| **Hip fracture** |  |  |  |
| Depressive symptoms score, continuous | 1.03(1.01,1.04) | 1.03(1.01,1.04) | 1.03(1.01,1.05) |
| Depressive symptoms status |  |  |  |
| No | Reference | Reference | Reference |
| Yes | 1.34(1.07,1.68) | 1.35(1.07,1.70) | 1.33(1.03,1.71) |
| Depressive symptom scores, quintile |  |  |  |
| 1(0-3） | Reference | Reference | Reference |
| 2(4-5) | 1.32(0.92,1.91) | 1.36(0.94,1.97) | 1.32(0.88,2.00) |
| 3(6-9) | 1.47(1.08,2.00) | 1.51(1.10,2.07) | 1.43(1.01,2.03) |
| 4(10-14) | 1.51(1.09,2.09) | 1.53(1.09,2.13) | 1.70(1.19,2.44) |
| 5(15-30) | 1.68(1.19,2.36) | 1.67(1.18,2.36) | 1.64(1.11,2.40) |
| **Fall** |  |  |  |
| Depressive symptoms score, continuous | 1.03(1.02,1.03) | 1.03(1.02,1.03) | 1.03(1.02,1.03) |
| Depressive symptoms status |  |  |  |
| No | Reference | Reference | Reference |
| Yes | 1.22(1.11,1.34) | 1.23(1.12,1.35) | 1.22(1.11,1.34) |
| Depressive symptoms score, quintile |  |  |  |
| 1(0-3） | Reference | Reference | Reference |
| 2(4-5) | 1.11(0.96,1.27) | 1.13(0.98,1.29) | 1.10(0.96,1.27) |
| 3(6-9) | 1.42(1.26,1.59) | 1.41(1.25,1.59) | 1.41(1.25,1.58) |
| 4(10-14) | 1.56(1.38,1.77) | 1.58(1.39,1.79) | 1.57(1.39,1.78) |
| 5(15-30) | 1.49(1.30,1.70) | 1.52(1.33,1.74) | 1.48(1.30,1.70) |

Model was adjusted for age, sex, residency, education, marital status, smoking status, drinking status, sleep duration and history of falls, BMI and physical comorbidities. Analysis 1: Excluding those currently have antidepressant; Analysis 2: Excluding those aged 80 years and above; Analysis 3: Excluding those fractured in the first wave.
